# Fostering Professional Identity Formation and Motivation for Joining Nursing and Midwifery Programmes among Undergraduate Nursing/Midwifery Students and Recent Graduates in Uganda

**DOI:** 10.5334/aogh.4435

**Published:** 2024-10-10

**Authors:** Scovia Nalugo Mbalinda, Kamoga Livingstone, Josephine Nambi Najjuma, Aloysius Mubuuke Gonzaga, Derrick Lusota, David Musoke, Samuel Owusu-Sekyere

**Affiliations:** 1 Department of Nursing, School of Health Science, College of Health Sciences, Makerere University, Kampala, Uganda; 2 Department of Nursing, Faculty of Medicine, Mbarara University of Science and Technology, Uganda; 3 Department of Radiology, School of Medicine, College of Health Sciences, Makerere University, Kampala, Uganda; 4 Department of Disease Control and Environmental Health, School of Public Health, College of Health Sciences, Makerere University, Kampala, Uganda; 5 African Forum for Research and Education in Health, Kumasi, Ghana

**Keywords:** Professional identity, Motivation, Nursing, Midwifery, Fostering

## Abstract

*Introduction:* The development of a strong professional identity is influenced by the motivation behind enrolling in a nursing or midwifery programme. Professional identity is a driving force that sustains the commitment of nurses and midwives to nursing/midwifery and their dedication to the well‑being of those they serve. This study evaluated Ugandan students’ reasons for enrolling in nursing and midwifery programmes. Furthermore, we investigated the nurse/midwifery practices that support professional identity creation in recent graduates and undergraduate nursing/midwifery students.

*Methods:* A mixed‑method research approach was employed amongst nursing/midwifery students of Makerere University and Mbarara University of Science and Technology and recent nursing/midwifery graduates from Mulago National and Mbarara Regional referral hospitals. We collected quantitative data from 173 participants, and for qualitative data, we conducted six focus group discussions among recent graduates and students of nursing/midwifery. We used descriptive statistics and thematic analysis to analyse the quantitative and qualitative data.

*Results:* Nearly all – 95.4% (165/173) – of the participants were motivated to undertake nursing/midwifery as their programme of study, and 94.2% (163/173) participants identified as nurses/midwives, all with an average score above 3. They also strongly agreed that they took up the programmes because they wanted to learn new things [111/173 (64.2%)] and considered nurses’ groups important [68.8% (119/173)]. Participants proposed measures to promote the formation of professional identity among students and graduates, including the improvement of clinical education, the phasing out of certain levels of practice, the empowerment and embedding of ethical principles, recognition and motivation, mentorship, leadership, career guidance and the inclusion of men and challenging of gender stereotypes.

*Conclusion:* Participants were motivated to work in nursing. The ways to promote professional identity included the improvement of clinical education, the phasing out of certain levels of practice, the empowerment and instillation of ethical principles, recognition and motivation, mentorship, leadership, career guidance and male inclusiveness and the challenging of gender stereotypes. Nursing and midwifery leadership needs to provide guidance, mentorship and empowerment; challenge gender stereotypes in nursing/midwifery practice; and give support while advocating for ethical practice.

## Introduction

Professional identity (PI) is a person’s understanding of their role and responsibilities, including their moral values [1]. Nursing students develop their moral competencies and PI primarily during their college/university years [2] Therefore, PI formation is a complex process that involves developing a sense of one’s role and responsibilities as a nurse or midwife, as well as one’s moral values and beliefs. As highlighted by Cornett et al. [3] PI formation is a dynamic and lifelong process influenced by various factors, including personal experiences, professional training and the broader social and cultural context. Thus, PI is important for individual and professional well‑being and the quality of healthcare delivery.

People are motivated to become nurses or midwives for various reasons, including a desire to serve others and God, a personal calling and the influence of family members and others, and through effective mentorship by other experienced nursing professionals [4]. According to reports from nurses, they aspire to work in a noble and compassionate profession caring for the sick and in the health industry [5]. Educators and regulators should understand the motivations of those who pursue nursing or midwifery careers to develop policies that foster professional identity formation in future healthcare workers. A strong professional identity is important for career satisfaction and retention [6].

The American Association of Colleges of Nursing (AACN), which provides the framework for baccalaureate and graduate nursing education in the USA, espoused professionalism as one of the 10 essential domains of nursing curricula, noting that professionalism involves ‘*formation and cultivation of a sustainable professional nursing identity*’ [7]. Simmonds et al. [8] support the AACN by stating that professional identity should be an explicit educational outcome of all nursing programmes. Wei et al. [9] and Traynor and Buus [10] suggested that many nursing students are unaware of their profession’s purpose, meaning and value and that their professional identity level decreases over time. This further highlights the importance of nurse and midwife educators facilitating professional identity development from the start of students’ training.

Nursing students’ professional identities significantly impact their willingness to stay in the nursing/midwifery field and how they transition from students to professional nurses [11] Evidence has shown that nursing/midwifery students with strong professional identities are more motivated to work in nursing and have a higher retention rate [11] Understanding the motivations that nurses and midwives have for entering the profession and fostering their professional identity has an implication for recruitment and retention, quality of care, patient satisfaction, safety and trust, workplace culture, career advancement, mental health and burnout prevention [6].

Understanding the motivations of nurses and midwives and fostering their professional identity is essential for the well‑being of patients, the effectiveness of healthcare delivery and the overall sustainability of the healthcare system. By recognising and supporting these motivations, healthcare organisations and educational institutions can contribute to a positive and thriving healthcare workforce [12]. Upon this background, we aimed to explore the motivations for joining nursing/midwifery and nurse/midwife practices fostering professional identity formation among undergraduate nursing/midwifery students and recent graduates in Uganda.

## Methods

### Study design

This study employed a concurrent mixed‑method approach research design among undergraduate nursing/midwifery students and recent graduates in Uganda.

### Study population

The study was conducted among current midwifery and nursing degree students and recent graduates at Mbarara University of Science and Technology (MUST) and Makerere University. The two universities are the oldest in Uganda, producing the largest group of nursing and midwifery graduates. They are both government‑owned universities. We also included recent midwives and nursing graduates practising nursing during their 1‑year mandatory training (internship) at Mulago National Referral Hospital (MNRH) and Mbarara Regional Referral Hospital (MRRH). Both hospitals receive interns from any of Uganda’s nine degree‑level nurse training institutions. MNRH is found in Kampala, the capital city, and serves as a national referral for all the hospitals in the country. It provides a range of services. MRRH is found in southwestern Uganda and serves about 14 districts and the countries neighbouring southwestern Uganda.

**Sample size estimation:** For the quantitative part of the study, we used the formula by Kish–Leslie for sample size calculation (citation of the formula) to obtain 384 students. This involved a finite population of 236 recent graduate and student nurses/midwives; hence, a final sample size of 173 participants was obtained after adjustment. For the qualitative data collection, participants were recruited until data saturation was attained.

### Inclusion criteria

This study included student nurses and midwives from Makerere University and Mbarara University of Science and Technology, and recent graduates from Mulago National and Mbarara regional referral Hospitals.

### Exclusion criteria

The study did not involve student nurses and midwives from Mbarara University or Makerere University or recent graduates from Mbarara Regional and Mulago National referral hospitals who did not feel well enough to take part in the activities.

### Data collection procedure

Participants in this study included recent graduates from Mulago National and Mbarara Regional Referral Hospitals, as well as student nurses and midwives from Makerere University and Mbarara University. Following the acquisition of signed informed consent, each participant filled out a questionnaire. The survey gathered personal data such as age, gender, marital status, occupation and reason for enrolling in a nursing or midwifery degree, in addition to the respondents’ professional identification ratings. The PI scores were collected using the Macleod Clark Professional Identity Scale (MCPIS‑9) – a nine‑item Likert scale [13]. We conducted six focus group discussions, with each focus group discussion composed of 8–12 participants who were recent graduates and students of nursing/midwifery. All the interviews were audio recorded, and notes were taken. We required the participants to identify nurse/midwife educator practices that fostered professional identity formation in nursing/midwifery.

Participants were also invited to contribute any other information they thought would be relevant to the discussion. The audio recordings of the interviews have a duration of 40–60 minutes each. Afterwards, the audio was transcribed verbatim. With the help of a professional notetaker, an experienced qualitative researcher led the focus group discussions.

### Data analysis

In our quantitative data analysis, we translated Likert scale responses into numerical values for the motivation and professional identity sections. Specifically, we assigned a value of 1 for “Completely unimportant” (or “Strongly disagree”), 2 for “Rather unimportant” (or “Disagree”), 3 for “Neutral,” 4 for “Important” (or “Agree”) and 5 for “Completely important” (or “Strongly agree”). To understand participant responses, we calculated the frequencies by determining how many participants provided an answer to a particular question, expressed as a percentage of the total number of participants (*N*/173 × 100, where *N* is the participant count). We calculated an average score for each participant’s response to a given question, with each response contributing to a total score out of 10. The average score was obtained by adding up the scores for all of the participants’ responses, out of a possible 10 points for each response. We used an average score threshold of 3 (a neutral midpoint for an average score with 5 as the highest) or higher to identify positive responses to questions. This threshold signifies that, on average, respondents tended to lean more towards agreement than disagreement with the statements, providing a clear indicator of the level of consensus on a given topic. These adjustments aimed to improve clarity and comprehension in our data analysis explanation.

The study team members who facilitated the interviews also provided debriefing notes and memos, in addition to transcripts of the audio‑recorded sessions. Interviews that were recorded were transcribed verbatim. The data analysis procedure started with open coding of the data (Level I), which involves going over the transcribed data from the interviews line by line to find the processes and contextual elements in the data. After comparing these variables or substantive codes with further data, categories were assigned (Level II). Groups were made out of coded data that seemed to show patterns or comparable information. To make sure the categories were mutually exclusive, they were then contrasted with one another. After that, categories were narrowed down by contrasting them to see how each one fit into a higher‑order category. To determine the main social processes or fundamental factors that explained the social scene, fewer categories were added (Level III). By creating additional speculative Level III codes, we were able to conceptualise the links between the three code levels [14].

### Ethical consideration

Before data collection, ethical approval was obtained from Makerere University College of Health Sciences, the School of Health Sciences Institutional Review Board (IRB; MAKSHSREC 2022‑415) and the Uganda National Council for Science and Technology (UNCST; HS2712ES) Administrative clearances were obtained from the administrations of hospitals and universities where we conducted the study. Written informed consent was obtained from the participants. The research team informed the participants at all levels about the survey and requested their voluntary participation. All respondents were assured confidentiality concerning the matters under discussion, as the interviews were conducted in special rooms. Audiotapes and notes did not contain participants’ personal identifiers and were kept in a locked file cabinet when not used.

### Demographic characteristics of the participants

From Table 1, we recruited 173 participants, achieving a 100% response rate for the quantitative part of the study, with 53.8% (93/173) of participants below the age of 25 years, more than half [59.5% (103/173)] female, 66.5% (115/173) single and 63% (109/173) students.

**Table 1:** Demographic characteristics of the participants.

AGE	*N* = 173	PERCENTAGE (%)
≤25 years	93	53.8
>25 years	80	46.2
**Sex**		
Male	70	40.5
Female	103	59.5
**Marital status**		
Single	115	66.5
Married	49	28.3
Cohabiting	8	4.6
Widowed	1	0.6
**Occupational status**		
Student	109	63.0
Recent graduate (intern)	68	39.3

### Motivation for studying nursing/midwifery

In Table 2, almost all the participants [95.36% (165/1732)] were motivated to undertake nursing/midwifery as their programme of study, with an average score above 3. They strongly agreed that they took up the programmes because they wanted to learn new things [111/173 (64.16%)] and they enjoyed studying nursing/midwifery [80/173 (46.24%)] because nursing/midwifery was an important life goal to them [106/173 (61.27%)]. They indicated that, as motivations for studying nursing/midwifery, others (parents, friends) obliging them to do so [49/173 (28.32%)] and wanting others to think they were smart [72/173(41.62%)] were completely unimportant.

**Table 2:** Frequency distribution for motivation for studying nursing or midwifery.

	COMPLETELY UNIMPORTANT	RATHER UNIMPORTANT	NEUTRAL	IMPORTANT	COMPLETELY IMPORTANT
	*N* (%)				
Because I want to learn new things	1 (0.58)	1 (0.58)	16 (9.25)	44 (25.43)	111 (64.16)
Because I’m supposed to do so	24 (13.87)	25 (14.45)	43 (24.86)	40 (23.12)	41 (23.70)
Because I enjoy studying nursing	7 (4.05)	3 (1.73)	17 (9.83)	66 (38.15)	80 (46.24)
Because this is an important life goal for me	2 (1.16)	2 (1.16)	12 (6.94)	51 (29.48)	106 (61.27)
Because studying nursing is an exciting thing to do	2 (1.16)	8 (4.62)	30 (17.34)	69 (39.88)	64 (36.99)
Because others (parents, friends) oblige me to do so	49 (28.32)	47 (27.17)	34 (19.65)	28 (16.18)	15 (8.67)
Because I would feel ashamed if I didn’t do so	45 (26.01)	44 (25.43)	45 (26.01)	22 (12.72)	17 (9.83)
Because I want others to think I am a good student/intern	52 (30.06)	45 (26.01)	39 (22.54)	21 (12.14)	16 (9.25)
Because studying nursing is fun	21 (12.14)	17 (9.82)	47 (27.17)	56 (32.37)	32 (18.50)
Because studying nursing is personally important to me	3 (1.73)	5 (2.89)	9 (5.20)	60 (34.68)	96 (55.49)
Because this represents a meaningful choice to me	1 (0.58)	2 (1.16)	16 (9.25)	69 (39.88)	85 (49.13)
Because that’s what others (parents, friends, etc.) expect me to do	38 (21.97)	54 (31.21)	42 (24.28)	20 (11.56)	19 (10.98)
Because that’s something others (parents, friends, etc.) force me to do	90 (52.02)	41 (23.70)	26 (15.03)	7 (4.05)	9 (5.20)
Because I want others to think I’m smart	72 (41.62)	42 (24.28)	30 (17.34)	19 (10.98)	10 (5.78)

### Professional identity

In Table 3, about 94.22% (163/173) participants identified as professional nurses/midwives, with a mean score above 3. They strongly agreed that they considered a nurses’ group important [68.79% (119/173)], that they identified with the nurses’ group [47.98% (83/173)] and that they were glad to belong to the nurses’ group [50.87% (88/173)].

**Table 3:** Frequency distribution for participants’ response on professional identity.

PROFESSIONAL IDENTITY	STRONGLY DISAGREE		DISAGREE		NEUTRAL		AGREE			STRONGLY AGREE	
	*N*	PERCENTAGE (%)	*N*	PERCENTAGE (%)	*N*	PERCENTAGE (%)	*N*	PERCENTAGE (%)	*N*	PERCENTAGE (%)
I am a person who considers a nurses’ group important.	4	2.31	0	0	4	2.31	46	26.59	119	68.79
I am a person who identifies with the nurses’ group.	0	0	3	1.73	11	6.36	76	43.93	83	47.98
I am a person who feels strong ties with the nurses’ group.	3	1.73	7	4.05	20	11.56	76	43.93	67	38.72
I am a person who is glad to belong to the nurses’ group.	3	1.73	5	2.89	12	6.94	65	37.57	88	50.87
I am a person who sees myself belonging to the nurses’ group.	2	1.16	5	2.89	18	10.40	82	47.40	66	38.15
I am a person who makes excuses for belonging to the nurses’ group.	62	35.83	75	43.35	19	10.98	10	5.78	7	4.04
I am a person who tries to hide belonging to the nurse’s group.	88	50.87	59	34.10	12	6.94	11	6.36	3	1.73
I am a person who feels held back by the nurses’ group.	76	43.93	68	39.31	11	6.36	14	8.09	4	2.31
I am a person who is annoyed to say I’m a member of the nurses’ group.	95	54.91	50	28.90	16	9.25	8	4.62	4	2.31
I am a person who would feel guilty for not belonging to the nurses’ group.	18	10.40	34	19.65	50	28.90	44	25.43	27	15.61

### Qualitative findings

In the qualitative part, we recruited 33 students and 26 recent graduates, of which 40 were females and 19 were males, aged 20–51 years (median age 25 years), who participated in six focus group discussions (FGDs; n = 59).

From the responses of the participants, the following key themes emerged: improving clinical education, better teaching methods and good clinical training, phasing out certain levels of nursing and midwifery education, improving the salary structure of nurses and midwives, interprofessional collaboration and leadership.

### Improving clinical education

The students value good clinical preceptorship in the ward because it improves clinical education quality and confidence to deliver, thereby enhancing their professional identity. Some of the participants mentioned this:

*The lecturers should follow students to ward and teach them practical skills that will help them change the attitude of students and become better nurses. (P08 FGD4 Student).*

*The people we are finding on the wards are the nurses; the university says they are the ones who are supposed to take us through, but they are not getting that time because when they see us on the ward, they see us as people who have come to help them and we know everything and yet we don’t know and yet these people since they are not like maybe they are not into that aspect of teaching someone systemically like step and step teaching, you find that this is affecting our learning as we don’t have the preceptors but I think if we really had them it will really improve on how we perceive nursing. (PO2 FGD4 Student).*

The participants also felt that, to be able to build and foster professional identity, there is a need to improve the teaching methods and provide good clinical training:

*I will talk about good clinical instruction; some schools have a way they handle their students when it comes to clinical instruction; if they have taught, let’s say, normal labour in class, they are going to allocate those students onward, and they will be only on the side where mothers that are low risk and those students will be strictly instructed on ground and she will make sure that whoever is allocated on that ward and have loopholes in terms of your practical output she will make sure that she iron out all those. (P03 FGD1 Recent graduate).*

*We should employ drills as ongoing drills; there are some hospitals that have adopted that practice, and each ward has a specific number of drills they run per month, and by drills, I mean just pose scenarios, then they pick randomly three people they say how would you interface with this scenario. These drills keep people’s minds awake concerning certain conditions and sometimes are about our psychological encounters. (P03 FGD1 Recent graduate).*

### Phasing out certain levels of nursing and midwifery education

The participants expressed that, for nursing and midwifery to foster professional identity, there is a need to phase out some levels of nursing and midwifery education, as indicated by the quotation below:

*We have a lot of levels of study, certificates, diplomas, and others, so they cause a lot of confusion and commotion in the hospital, so if they are to phase out some of them or most of them except maybe for diploma where the minimum level of entry into nursing is a diploma. They make the nursing profession lose meaning and quality; if that happens and in the clinical area, we have bachelors and are the ones working there, they know what they learnt, and they have the knowledge to teach us and guide us, they know what they are supposed to do, and so they teach us that particular thing. (P09 FGD6 student).*

### Empowering and instilling ethical principles

The participants thought instilling nursing and ethical values in the students and practising nurses and midwives would go a long way towards fostering professional identity. They believed this would improve self‑esteem and autonomy among nurses. It would also enable specialisation within the profession.

*Educate the nurses, starting from the certificate, that nursing is a different profession from being a Dr, and you are not supposed just to follow a doctor like a puppy or follow whoever you find like a puppy; you are supposed to be yourself and make your decisions to be autonomous (P03 FGD1 recent graduate).*

*I appreciate my lecturers because they have always taught us to be autonomous, that you are not supposed to just go with whatever they are telling you, doctors. They are also human beings, and they make mistakes, and they have empowered us to respectfully challenge whatever mistake they have made (P03 FGD1 recent graduate).*

### Recognition and motivation of nurses and midwives

The participants mentioned staying and understanding the patients longer than any healthcare provider. It is very important to motivate the nurses and the midwives.

*The government should increase the salary for the nurses and midwives because you find that nurses do a lot of work in the hospital, and they are given less, so they are less motivated, and they end up coming late for work. (P04 FGD6 Student).*

*The government somehow is playing a role in all this inferiorism in nursing. I feel like they are judging the work by the amount of money they pay us. You find that here in Uganda, in my perspective, I feel the value of my work is somehow portrayed by the money I receive. (P13 FGD1 Recent graduate).*

### Mentorship

The participants believe mentorship is pivotal in developing the professional identity of nurses and midwives. They believe this helps them understand their roles, values and responsibilities in the nursing and midwifery profession.

*As student nurses, we need to embrace the core values of the nursing profession and try to engage with our peers or mentors in the profession to build ourselves to become better nurses. (P01 FGD05 student).*

*However much we have different barriers, we must beat all these odds and set the best example there as being a good nurse starts with an individual; you must work on yourself first before you talk about others conduct themselves well be that example that a junior student nurse will look at and be like I want to be like her. So, I think we can start with being a good example, embracing the value, attributes, and good qualities of a good nurse (P01 FGD05 student).*

### Leadership

The participants believed there is a need for the nursing and midwifery leadership to be involved in fostering professional identity within nursing and midwifery schools. Likewise, if the students develop a strong professional identity, it will enable them to be effective leaders in the future.

*The nursing council should supervise most of the training schools such that they make sure most of the nursing values are instituted in the student nurses and midwives (P08 FGD05 student).*

*Most nurses don’t want to associate themselves as nurses/midwives because the picture outside is for you to join nursing is that you must have failed, so that was a picture way back, but currently, it’s no longer the same to join nursing; you must have performed well… This is the role of the nurses’ council as they regulate practice when we graduate and go back to our places of work. It’s quite challenging. So, the nurses’ council and other relevant bodies need to work on it (P1O FGD01 Recent graduate).*

### Career guidance

Participants think that it would be good to have career guidance in high schools and as soon as they join the nursing schools. Most of them do not know what it entails to be a nurse or midwife at the beginning of the programme.

*Children must be inspired that nursing is a good job; it’s not a second choice, and you can do it and be a good nurse and do exceptionally good things. So, I think children must be inspired by career guidance. People already in nursing can go to schools; you know I am doing this; that’s what kids need to hear (PO5 FGD01 Recent graduate).*

*There’s no career guidance; you are there and don’t know if you should upgrade. That’s why when most people finish their diploma in nursing, they start meandering, let me go and double train because you can’t identify yourself, you are just getting lost, but you go for a bachelor’s degree and continue, your self‑esteem goes up, you start valuing yourself as a nurse, you know the values as a nurse (P01 FGD01 Recent graduate).*

### Male inclusiveness and gender stereotypes

Participants agreed that promoting male inclusiveness in nursing and midwifery is important. Male inclusiveness can help them develop a strong professional identity.

*Leadership in nursing also needs male involvement. Leadership is mostly dominated by ladies as you know, sometimes you know men and women are different. Have different attributes of how we see things, so I think we can also put some good numbers of men in leadership of nursing (P12 FGD01 Recent graduate).*

*There is a need for gents to be represented that also stems from practice on how we call ourselves as midwives and nurses; we use a word like ‘sister’ that is not inclusive at all. I have encountered gents being bullied so many times; they are calling this man sister. It kind of demoralises them. OK, we accept that it’s mostly seen as a lady’s profession, but we have evolved past that. I think we need to get different ways of addressing each other and a much more respectful way of addressing ourselves on the ward (P04 FGD01 Recent graduate).*

## Discussion

In this study, we examined the nurse/midwife practices that support the development of professional identity (PI) among undergraduate nursing/midwifery students and recent graduates in Uganda, as well as the reasons why they enrolled in these programmes. The major themes for fostering professional identity that emerged included the phasing out of certain levels of practice, the improvement of clinical education, the empowerment and instillation of ethical principles, recognition and motivation, mentorship, leadership, career guidance and male inclusivity and gender stereotypes. The participants were motivated to pursue careers in nursing and midwifery.

In this study, we found out that most of the participants were motivated to undertake nursing/midwifery as their programme of study, with the average score of the total participants’ responses being above 3. We further found that the most common reasons for nursing/midwifery students and recent graduates choosing nursing/midwifery were a desire to learn new things, the love of studying nursing/midwifery and nursing/midwifery being an important life goal. These findings are consistent with previous research [15, 16] which found that the most common reasons for entering nursing were a desire to help others, an interest in science and healthcare and a belief that nursing is a rewarding career. Since students/recent graduates choose to study nursing/midwifery for intrinsic reasons such as the desire to learn new things and the love of nursing, which is a positive sign for the profession, we had an important finding from this study, which showed that external pressures, such as the expectations of others such as parents or friends, were relatively less important motivators for nursing/midwifery students/recent graduates. This is similar to Armstrong et al. [17] who also found that external motivation, such as financial rewards and prestige, was not unimportant for nurses’ satisfaction and commitment.

Furthermore, a significant percentage of participants were classified as professional nurses or midwives. They enthusiastically agreed that a nurses’ group is vital, identified with the organisation and were happy to be a part of it. This is consistent with earlier research, such as that conducted by Browne et al. [18] which showed that nurses who had a strong sense of identity as professionals are more likely to be content in their roles, enjoy job retention and deliver superior patient care.

Nursing and midwifery students and recent graduates can develop a strong sense of self as professionals by investing in clinical education, employing effective teaching strategies and offering supporting clinical training with clinical mentors and faculty presence. Thus, the nursing and midwifery professions may be able to flourish as a result. Our results are consistent with those of Clements et al., Murdock et al. and Johnson et al. [19–21] who discovered that students were more likely to form a strong professional identity if they got excellent mentor and faculty support. This implies that preserving the future of the profession can be greatly aided by nursing and midwifery instructors who place a high priority on clinical teaching and offer sufficient support.

The participants expressed their support for the gradual elimination of specific nursing and midwifery education levels, pointing out that the negative attitudes and low self‑esteem of nurses belonging to these cadres had damaged their reputation as professionals. They contended that this might negatively affect how students and recent hires in the field build their professional identities. An increasing body of research indicates that nurses who have positive attitudes and high self‑esteem are more likely to have a strong sense of self as professionals, which lends credence to this claim. For instance, a study by Serafin et al. [22] discovered that nurses who had higher self‑esteem were more likely to strongly identify with the nursing profession and to report feeling competent and confident in their work. Another study [23] found that nurses with positive attitudes were more likely to be satisfied with their work and to have higher job morale. These findings suggest that phasing out certain levels of nursing and midwifery education could help improve the nursing profession’s overall professional image and promote the development of strong professional identities among nurses.

Empowering nurses and instilling ethical principles makes nurses/midwives recognise the ethical implications of all nursing/midwifery actions. Nurses and midwives who adhere to ethical principles have a well‑defined professional identity and are better equipped to provide high‑quality care and advocate for their patients [24] Professional development for nurses and midwives enhances knowledge, skills and expertise; this builds the nurse’s confidence and competence and promotes self‑reflection and self‑improvement, thus reinforcing professional identity and participating in research and evidence‑based practice [25] Professional development helps nurses and midwives keep abreast with current trends and best practices, improving patient outcomes and enhancing their professional identity [25]. There is a need for leadership to promote continuing education and lifelong learning and increase participation in professional organisations and networks.

Participants in this study asserted that their work and contribution to the health sector are evaluated primarily in terms of their salary. They proposed that improving the salary structure of nurses and midwives would bolster their motivation and affirm their professional identity. This finding is supported by research demonstrating that salary is a critical determinant of job satisfaction and professional identity. For instance, a study by Rekisso [26] found that nurses who earned higher salaries were more likely to report feeling satisfied with their work and valued as professionals. Furthermore, a study [27] discovered that nurses with a strong professional identity were more likely to be content with their salary and believe they were fairly compensated. This notwithstanding, increasing the salary structure for nurses and midwives could further enhance job satisfaction, professional identity and overall well‑being; in addition, it was found that nurses who felt they were being adequately compensated and had a strong sense of identity as professionals were more likely to be satisfied with their wage. Despite this, raising the pay scale for nurses and midwives could improve their general well‑being, sense of professional identity and job satisfaction even more.

To support professional identity in nursing, mentoring is essential. In addition to fostering confidence and self‑esteem, developing critical thinking and decision‑making skills and facilitating career advancement and professional growth, faculty and preceptor mentoring from classes allows students to process clinical knowledge, skills, values and attitudes that need clarification [28]. Novice nurses and midwives benefit professionally from having a strong mentoring connection [29]. Research has demonstrated that mentoring can help students recognise the positive and negative qualities of their mentors and develop their professional identities. This can help advance the fields of nursing and midwifery in the future [30, 31] Nurse mentors guide novices along the road of professional progress, prospects within the field and potential happiness. They serve as professional role models for novices. Thus, to promote professional identity, mentorship programmes and support networks must be put in place.

A key element in developing a professional identity is leadership. By evaluating their level of proficiency and offering ways to support their growth and development, nurse and midwife leaders can assist nurses and midwives in establishing their professional identities [32]. Leadership roles for nurses and midwives provide a special opportunity to uphold professional identity and provide an example for behaviour [32]. To support professional identity, leadership is required. This includes offering mentorship and direction, fostering autonomy and decision‑making, encouraging cooperation and teamwork and appreciating and acknowledging the contributions made by nurses.

Career guidance can help nurses and midwives develop professional identity by providing guidance and support in career development. Career guidance helps nurses and midwives develop a sense of belonging and purpose, improving their well‑being, work retention and job satisfaction, promoting safe, quality patient care [33]. Furthermore, it strengthens their professional identity and keeps them abreast of the most recent developments and best practices in nursing [33]. There is a need to provide career guidance to students and newly qualified nurses and midwives so that they can identify their passions and interests in the nursing profession and shape their professional identity.

Stereotypes about gender have an impact on those who are considering a career in nursing or midwifery, as well as on women and men who work in these fields [34]. Negative stereotypes associated with nursing and midwifery professionals can impact male nurses’ professional competencies and masculinity [35]. Male inclusiveness is important in fostering professional identity. Many studies have found male nursing students and male nurses to perceive social prejudice against them [36, 37] Male nursing students and male nurses may face challenges owing to being a minority in a female‑oriented profession [34]. This may affect their professional identity formation, but they also have greater opportunities for professional advancement. There is a need to tackle stereotypes and raise public awareness using gender‑inclusive language and strategies for recruitment and ensuring gender diversity in nursing teams.

In conclusion, the participants were inspired to pursue careers in nursing and midwifery. Enhancing clinical education; phasing out particular practice levels; empowering and embedding ethical ideals; providing acknowledgement and motivation, mentorship, leadership and career guidance; ensuring male inclusivity; and challenging gender stereotypes are a few strategies to promote professional identity. In addition to promoting ethical behaviour, leadership in nursing and midwifery should offer direction, mentorship, and empowerment and address gender stereotypes in the profession and support.

### Strength and limitation

To the best of our knowledge, this study is the first to investigate how nurses and nursing students might develop their professional identities in low‑resource settings. This is important because, in many circumstances, nurses serve as the backbone of the healthcare system. The participants’ detailed and varied accounts of their experiences gave rise to a thorough understanding of clinical learning. Because of the high calibre of data gathering and analysis, the study is extremely rigorous, and the conclusions are reliable. Notwithstanding these advantages, the results are applicable to comparable situations even though they might only apply to the students’ viewpoint.

## Figures and Tables

**Table:** 

FGD	Focus group discussion
MRRH	Mbarara Regional Referral hospital
MNRH	Mulago National Referral Hospital
PI	Professional identity
UNCST	Uganda National Council for Science and Technology

## Data Availability

The datasets used and/or analysed during the current study are available from the corresponding author on reasonable request.
